# Activation of PKA, p38 MAPK and ERK1/2 by gonadotropins in cumulus cells is critical for induction of EGF-like factor and TACE/ADAM17 gene expression during *in vitro *maturation of porcine COCs

**DOI:** 10.1186/1757-2215-2-20

**Published:** 2009-12-24

**Authors:** Yasuhisa Yamashita, Mitsugu Hishinuma, Masayuki Shimada

**Affiliations:** 1 School of Veterinary Medicine, Faculty of Agriculture, Tottori University, 4-101 Koyamachou-minami, Tottori, 680-8553, Japan; 2 Graduate School of Biosphere Science, Hiroshima University, 1-4-4 Kagamiyama, Higashi-Hiroshima, 739-8528, Japan

## Abstract

**Objectives:**

During ovulation, it has been shown that LH stimulus induces the expression of numerous genes via PKA, p38 MAPK, PI3K and ERK1/2 in cumulus cells and granulosa cells. Our recent study showed that EGF-like factor and its protease (TACE/ADAM17) are required for the activation of EGF receptor (EGFR), cumulus expansion and oocyte maturation of porcine cumulus-oocyte complexes (COCs). In the present study, we investigated which signaling pathways are involved in the gene expression of EGF-like factor and in *Tace/Adam17 *expression in cumulus cells of porcine COC during *in vitro *maturation.

**Methods:**

*Areg*, *Ereg*, *Tace/Adam17*, *Has2*, *Tnfaip6 *and *Ptgs2 *mRNA expressions were detected in cumulus cells of porcine COCs by RT-PCR. Protein level of ERK1/2 phosphorylation in cultured cumulus cells was analyzed by westernblotting. COCs were visualized using a phase-contrast microscope.

**Results:**

When COCs were cultured with FSH and LH up to 2.5 h, *Areg*, *Ereg *and *Tace/Adam17 *mRNA were expressed in cumulus cells of COCs. *Areg*, *Ereg *and *Tace/Adam17 *gene expressions were not suppressed by PI3K inhibitor (LY294002), whereas PKA inhibitor (H89), p38 MAPK inhibitor (SB203580) and MEK inhibitor (U0126) significantly suppressed these gene expressions. Phosphorylation of ERK1/2, and the gene expression of *Has2*, *Tnfaip6 *and *Ptgs2 *were also suppressed by H89, SB203580 and U0126, however, these negative effects were overcome by the addition of EGF to the medium, but not in the U0126 treatment group.

**Conclusion:**

The results showed that PKA, p38 MAPK and ERK1/2 positively controlled the expression of EGF-like factor and TACE/ADMA17, the latter of which impacts the cumulus expansion and oocyte maturation of porcine COCs via the EGFR-ERK1/2 pathway in cumulus cells.

## Background

In mammals, luteinizing hormone (LH) stimulation induces morphological and physiological changes in granulosa cells and cumulus cells, causing them to progress to the ovulation process [1]. During this period, cumulus cells expressed cumulus expansion-related genes, *Hyaluronan synthase 2 *(*Has2*) [2,3], *Tumor necrosis factor α-induced protein 6 *(*Tnfaip6*) [4,5], and *Pentraxin 3 *(*Ptx3*) [6,7], which is necessary for the synthesis and stability of hyaluronan-rich extracellular matrix. In *Tnfaip6 *null mice [5] or *Ptx3 *null mice [7], number of ovulated oocytes decreased and *in vivo *fertilization was completely interrupted, suggesting that cumulus expansion was essential for both ovulation and fertilization processes.

It is known that since LH receptor (*Lhcgr*) is mainly expressed in granulosa cells, the EGF-like factor produced in granulosa cells by LH surge acts on cumulus cells to induce cumulus expansion. Some factors were introduced to transmit the LH signal from granulosa cells to cumulus cells. For example, prostagrandin E2 (PGE2) that produced from granulosa cells and cumulus cells by prostagradin synthase 2 (PTGS2) was required for induction of *Has2 *and *Tnfaip6 *gene, cumulus expansion and oocyte meiotic resumption [8]. The EGF-like factors, Amphiregulin (AREG), Epiregulin (EREG) and β-cellulin (BTC) were also recently reported as potent factor. The EGF-like factor was induced by LH stimuli in granulusa cells, and EGF receptor (EGFR) was localized on cumulus cells [9-11]. When mouse COCs were cultured with AREG, *Has2*, *Tnfaip6 *and *Ptgs2 *were expressed in cumulus cells. TACE/ADAM17, the cleavage enzyme of EGF-like factor to soluble forms, was also expressed in porcine granulosa cells *in vivo *in response to hCG administration [12]. Thus, *in vivo *during the ovulation process, LH induces EGF-like factor expression in granulosa cells and the release of the EGF domain by TACE/ADAM17 acts on cumulus cells, which induce cumulus expansion.

In *in vitro *maturation of oocytes, COCs were recovered from antral follicles and cultured with FSH and/or LH. We previously showed that FSH and LH up-regulate EGF-like factor and *Tace/Adam17 *mRNA expression, and gonadotropins-induced cumulus expansion and oocyte maturation of porcine COCs were suppressed by EGF receptor tyrosine kinase inhibitor or TACE/ADAM17 inhibitor [13]. The results suggested that FSH- and LH-induced cumulus expansion was dependent on the expression and functions of EGF-like factors. Although the regulation of EGF-like factor expression in cancer cell lines has been reported [14,15], the mechanisms of EGF-like factor and TACE/ADAM17 expression in cumulus cells cultured with FSH and/or LH have remained unclear during *in vitro *maturation of porcine COCs.

The binding of FSH and/or LH in granulosa cells to its own receptors led to rapidly and nongenomic activation of PKA, p38 MAPK, and PI3K in a cAMP-dependent manner [16] and of ERK1/2 via the SRC/RAS-dependent pathway [17]. In mice, since each inhibitor of PKA, p38 MAPK, PI3K or ERK1/2 suppressed the expression of cumulus expansion-related gene [10,18,19], cumulus expansion [18,19] or meiotic maturation of oocyte [20], we estimated that these signaling pathways induced by gonadotropin overlap the EGF-like factor-EGFR pathway, which induces full cumulus expansion and oocyte maturation.

In this study, to clarify the intracellular pathway involved in EGF-like factor and *Tace/Adam17 *expression in cumulus cells, we examined the effect of PKA inhibitor (H89), p38 MAPK inhibitor (SB203580), PI3K inhibitor (LY294002) and MEK inhibitor (U0126) on *Areg*, *Ereg *and *Tace/Adam17 *expression in cumulus cells during *in vitro *maturation of porcine COCs. Additionally, we investigated the effect of these drugs on ERK1/2 phosphorylation, cumulus expansion and oocyte meiotic resumption in pig.

## Methods

### Materials

High purified porcine FSH and porcine LH were gifts from the National Hormone and Pituitary Program (Rockville, MD, USA). Fetal calf serum (FCS) was obtained from Invitrogen (Carlsbad, CA, USA). Oligonucleotide poly- (dT) was purchased from Amersham Pharmacia Biotech (Newark, NJ, USA). Avian myeloblastosis virus reverse transcriptase and Taq DNA Polymerase were from Promega (Madison, WI). Routine chemicals and reagents were obtained from Nakarai Chemical Co. (Osaka, Japan) or Sigma (Sigma Chemical Co., St. Louis, MO, USA).

### In vitro culture of porcine COCs

Isolation of porcine COCs was described previously [21]. Briefly, porcine ovaries were recovered from 5- to 7-month-old prepubertal gilts at a local slaughterhouse. COCs were collected from the surfaces of intact healthy antral follicles measuring from 3 to 5 mm in diameter. The 20 COCs were cultured up to 40 h with both 20 ng/ml highly purified porcine FSH (NIDDK, Torrance, CA) and 500 ng/ml porcine LH (NIDDK). The maturation medium was modified NCSU37 [22] supplemented with 10% (v/v) FCS (Gibco BRL, Grand Island, NY) and 7 mM Taurine (Sigma St Louis, MO). At selected time intervals, COCs were collected for RNA and protein isolation.

The assessment of cumulus expansion was observed using phase-contrast microscopy (Olympus IMT2, Olympus, Tokyo Japan) and a 10× objective.

### Treatment with inhibitors

In the case of treatment with each specific inhibitor, namely PKA, H89 (10 uM) (Sigma), p38 MAPK, SB203580 (20 uM) (Sigma), PI3K, LY294002 (20 uM) (Sigma) or MEK, U0126 10 uM (Sigma), COCs were cultured for 0, 2.5, 5, 10, 20 or 40 h with each of these inhibitors. H89 was dissolved in maturation medium at 10 mM and stored at -20°C. SB203580, LY294002 and U0126 were dissolved in dimethylsulfoxide (DMSO) at 20 mM and 10 mM, respectively, and stored at -20°C. The final concentration of each compound (as described above) was obtained by dilution (1:1000) with the maturation medium. The final concentration of vehicles (DMSO) was 0.1% (vol/vol), which did not affect the function of cumulus cells during meiotic resumption of porcine oocytes [23].

### RNA isolation

After cumulus cells were cultured, they were washed three times in PBS. Total RNA was extracted from cumulus cells using the SV Total RNA Isolation System (Promega) according to the instruction manual, and dissolved in nuclease-free water. The final RNA concentrations were determined by absorbance using a spectrophotometer.

### RT-PCR

RT-PCR analyses were performed as previously described [22]. Briefly, total RNA was reverse-transcribed using 500 ng poly-dT and 0.25 U of avian myeloblastosis virus-reverse transcriptase at 42°C for 75 min and 95°C for 5 min. PCR conditions were set as follows: cDNA was amplified for X cycles (Table 1) of denaturation at 94°C for 30 sec, primer annealing at Y°C (Table 1) for 1 min, and extension at 68°C for 1 min, with a final extension step of 7 min at 68°C. *β-actin *was used as a control for reaction efficiency and variations in concentration of mRNA in the original RT reaction. The amplified products were analyzed by electrophoresis on 2% agarose gels. The intensity of the objective bands was quantified by densitometric scanning using a Gel-Pro Analyzer. Specific primer pairs were selected and analyzed as indicated in Table 1.

**Table 1:** List of primers employed for RT-PCR

G**ene**	Forward Primer	Reverse Primer	Anneling temprature (X)	C**ycle (Y)**
*β-actin*	5'-CTA CAA TGA GCT GCG TGT GG-3'	5'-TAG CTC TTC TCC AGG GAG GA-3'	58	31
*Areg*	5'-CAC CAG AAC AAA AAG GTT CTG TC-3'	5'-AAG TCC ATG AAG ACT CAC ACC AT-3'	58	35
*Ereg*	5'AAG ACA ATC CAC GTG TGG CTC AAG-3'	5'-CGA TTT TTG TAC CAT CTG CAG AAA-3'	58	35
*Tace/Adam17*	5'-GAC ATG AAT GGC AAA TGT GAG AAA C-3'	5'-AGT CTG TGC TGG GGT CTT CCT GGA-3'	58	34
*Has2*	5'-GAA TTA CCC AGT CCT GGC TT-3'	5'-GGA TAA ACT GGT AGC CAA CA-3'	54	35
*Tnfaip6*	5'-TCA TAA CTC CAT ATG GCT TGA AC-3'	5'-TCT TCG TAC TCA TTT GGG AAG CC-3'	54	32
*Ptgs2*	5'-CTG CCG TGT CGC TCT GCA CTG-3'	5'-TCA TAA CTC CAT ATG GCT TGA AC-3'	58	35

### Western blot analysis

Cumulus cells were lysed in Laemmli sample buffer and protein extracts were stored at -80°C until use. After denaturing by boiling for 5 min, 10 ul of each samples containing equal amounts of protein (10 ug) was separated by SDS-PAGE on 10% polyacrylamide gel, then transferred onto PVDF membrane (GE Healthcare). The membrane was blocked with 5% (w/v) nonfat dry milk (GE Healthcare) in PBS. Primary antibodies were added in 2.5% (w/v) nonfat dry milk in 0.1% (v/v) Tween 20 (Sigma)/PBS (PBS-T), and incubated overnight at 4°C. Anti-phospho-ERK1/2 and β-ACTIN were purchased from Cell Signaling Technology, Inc (Beverly, MA) and diluted at 1:2,000 or 1:10,000, respectively. After four washes in PBS-T, the membranes were incubated for 1 h with a 1:2,000 dilution of goat anti-rabbit IgG HRP-linked antibody (Cell Signaling Technology, Inc) in 2.5% (w/v) nonfat dry milk in PBS-T at room temperature. After five washes of 10 min each with PBS-T, peroxidase activity was visualized using the ECL Western blotting detection system (GE Healthcare) according to the manufacturer's instructions.

### Statistical analysis

Statistical analyses of all data from three or four replicates for comparison were carried out by one-way ANOVA followed by Duncan's multiple-range test (Statview; Abacus Concepts, Inc., Berkeley, CA). All percentage data were subjected to arcsine transformation before analysis.

## Results

### Effect of each specific inhibitor of PKA, p38 MAPK, PI3K and MEK on the gonadotropin-induced Areg, Ereg and Tace/Adam17 mRNA expression during in vitro maturation of porcine COCs

**Figure 1:**
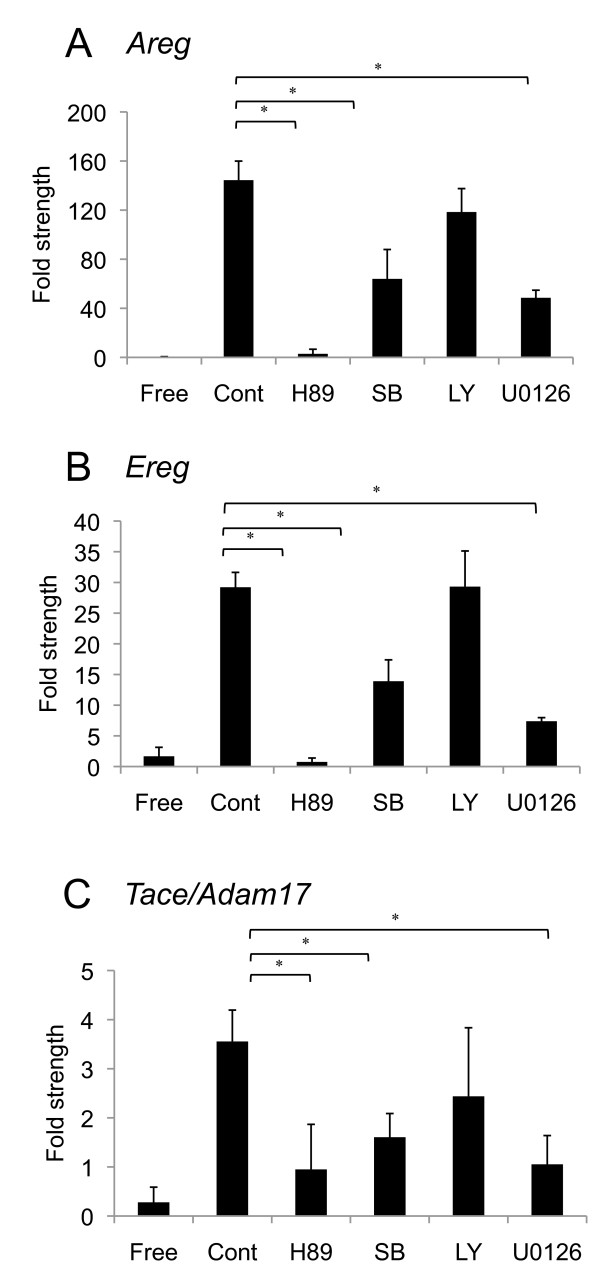
**Effect of H89, SB203580, LY294002 or U0126 on *Areg*(A), *Ereg*(B) or *Tace/Adam17*(C) mRNA**. For reference, the 0 h COC value was set as 1 and the data presented as the fold strength. Values are mean +/- SEM of 3 replicates. *: The significant differences were observed as compared with that in COCs cultured with FSH and LH for 2.5 h. The respective value of among *Areg*, *Ereg*, and *Adam17 *mRNA were normalized according to those of *β-actin *mRNA to evaluate arbitrary units of the relative abundance of the targets. Free: COCs were cultured without FSH and LH for 2.5 h; Cont: COCs were cultured with FSH and LH for 2.5 h; H89: COCs were cultured with FSH, LH and H89 for 2.5 h; SB: COCs were cultured with FSH, LH and SB203580 for 2.5 h; LY: COCs were cultured with FSH, LH and LY294002 for 2.5 h; U0126: COCs were cultured with FSH, LH and U0126 for 2.5 h

COCs were cultured with FSH, LH and/or PKA inhibitor (H89), p38 MAPK inhibitor (SB203580), MEK inhibitor (U0126) or PI3K inhibitor (LY294002) for 2.5 h. The results showed that high levels of *Areg*, *Ereg *and *Tace/Adam17 *mRNA were observed when COCs were cultured with FSH and LH, and that the levels were not affected by LY294002 (Figure 1A, B, C). However, treatment with H89, SB203580 or U0126 significantly decreased these mRNA expression levels in cumulus cells (Figure 1A, B, C).

### Effect of each specific inhibitor of PKA, p38 MAPK and MEK on ERK1/2 phosphorylation in cumulus cells during in vitro maturation of porcine COCs

**Figure 2:**
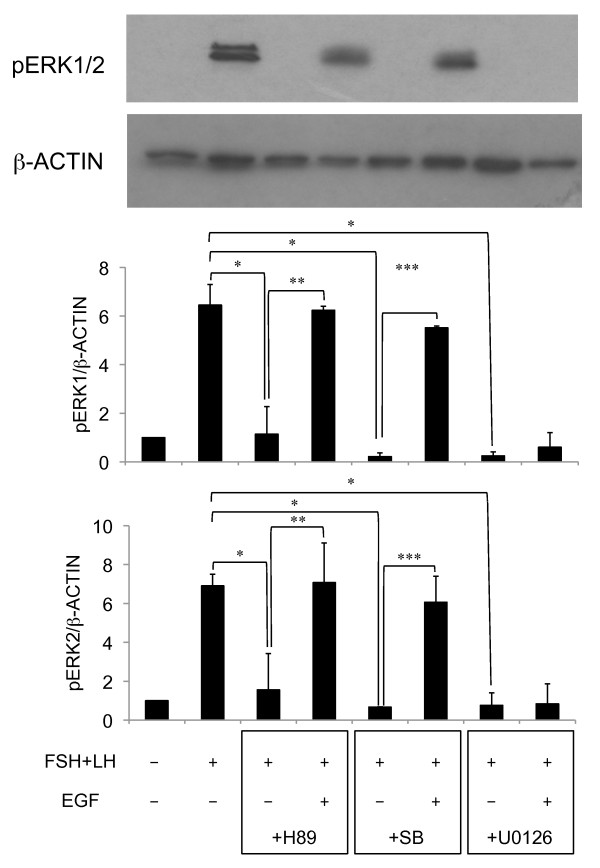
**Effect of H89, SB203580 or U0126 on ERK1/2 phosphorylation in cumulus cells**. For reference, the COC that were cultured without FSH and LH for 5 h value was set as 1 and the data presented as the fold strength. Values are mean +/- SEM of 3 replicates. *: The significant differences were observed as compared with that in COCs cultured with FSH and LH for 5 h. **: The significant differences were observed as compared with that in COCs cultured with FSH, LH, H89 and EGF for 5 h. ***: The significant differences were observed as compared with that in COCs cultured with FSH, LH SB230580 and EGF for 5 h. The respective value of protein levels of ERK1/2 phosphorylation were normalized according to those of β-ACTIN to evaluate arbitrary units of the relative abundance. FSH(-): COCs were cultured without FSH and LH for 5 h; FSH(+): COCs were cultured with FSH and LH for 5 h; EGF(-): COCs were cultured without EGF for 5 h; EGF(+): COCs were cultured with EGF for 5 h; +H89: COCs were cultured with H89 for 5 h; +SB: COCs were cultured with SB203580 for 5 h; +U0126: COCs were cultured with U0126 for 5 h

When COCs were cultured with FSH and LH for 5 h, the phosphorylation status of ERK1/2 was detected. The addition of H89 or SB20580 to the medium significantly decreased the intensity of bands as compared with those in cumulus cells of COCs cultured without any inhibitor, and these inhibitory effects were overcome by addition of EGF (Figure 2). Treatment with U0126 also significantly suppressed ERK1/2 phophorylation in cumulus cells as compared with those in cumulus cells of COCs cultured without U0126; however, the addition of EGF to U0126-contained medium did not affect the phosphorylation.

**Figure 3:**
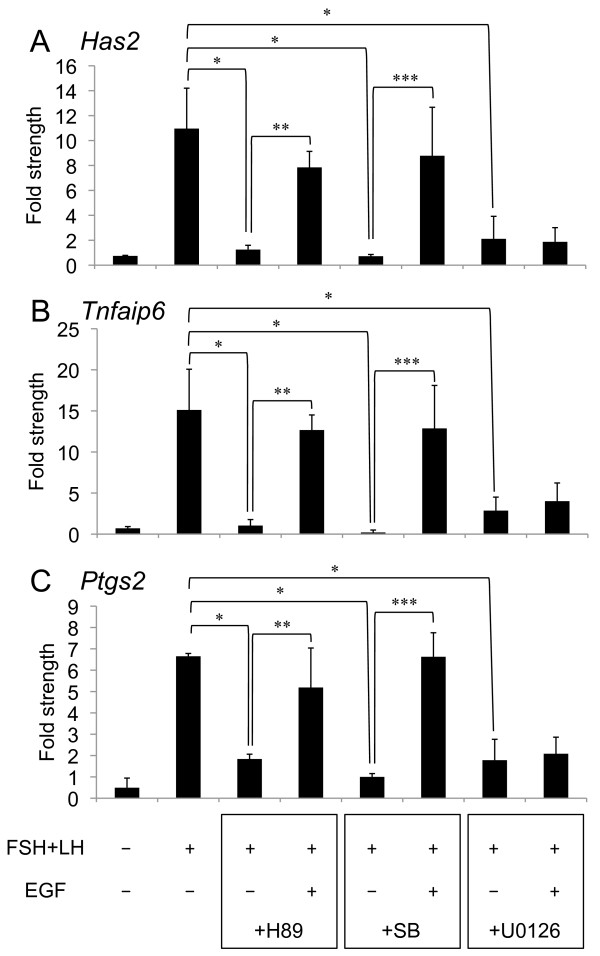
**Effect of H89, SB203580 or U0126 on expression of *Has2*(A), *Tnfaip6*(B) or *Ptgs2*(C) mRNA**. For reference, the 0 h COC value was set as 1 and the data presented as the fold strength. Values are mean +/- SEM of 3 replicates. *: The significant differences were observed as compared with that in COCs cultured with FSH and LH for 10 h. **: The significant differences were observed as compared with that in COCs cultured with FSH, LH, H89 and EGF for 10 h. ***: The significant differences were observed as compared with that in COCs cultured with FSH, LH, SB230580 and EGF for 10 h. The respective value of among *Has2*, *Tnfaip6 *and *Ptgs2 *mRNA were normalized according to those of *β-actin *mRNA to evaluate arbitrary units of the relative abundance of the targets. FSH(-): COCs were cultured without FSH and LH for 10 h; FSH(+): COCs were cultured with FSH and LH for 10 h; EGF(-): COCs were cultured without EGF for 10 h; EGF(+): COCs were cultured with EGF for 10 h; +H89: COCs were cultured with H89 for 10 h; +SB: COCs were cultured with SB203580 for 10 h; +U0126: COCs were cultured with U0126 for 10 h

High levels of *Has2*, *Tnfaip6 *and *Ptgs2 *expression were observed in cumulus cells of COCs cultured for 10 h with FSH and LH as compared with those cultured without FSH and LH. The addition of H89, SB20580 or U0126 significantly suppressed FSH- and LH-induced *Has2*, *Tnfaip6 *and *Ptgs2 *expressions (Figure. 3A, B, C). Although the lower level of these gene expressions resulting from the addition of U0126 was not overcome by the addition of EGF, the addition of EGF to H89- or SB203580-containning medium overcame the negative effect of each inhibitor on these gene expressions (Figure. 3A, B, C).

### Effect of each specific inhibitor of PKA, p38 MAPK and MEK on meiotic resumption of oocytes and cumulus expansion during in vitro maturation of porcine COCs

**Figure 4:**
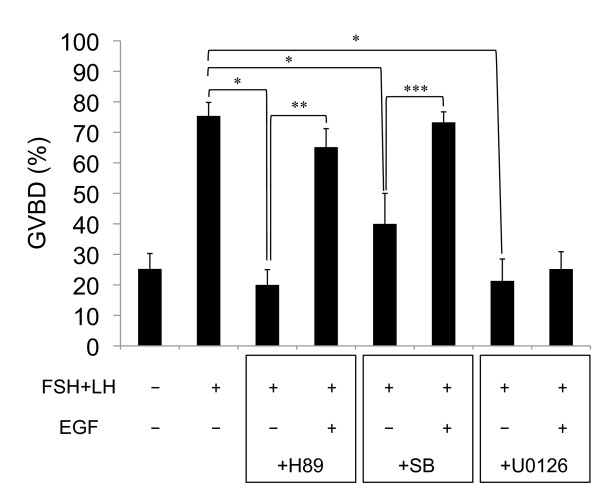
**Effect of H89, SB203580 or U0126 on rate of oocyte exhibiting GVBD**. Values are mean +/- SEM of 3 replicates. *: The significant differences were observed as compared with that in COCs cultured with FSH and LH for 20 h. **: The significant differences were observed as compared with that in COCs cultured with FSH, LH, H89 and EGF for 20 h. ***: The significant differences were observed as compared with that in COCs cultured with FSH, LH, SB230580 and EGF for 20 h. FSH(-): COCs were cultured without FSH and LH for 20 h; FSH(+): COCs were cultured with FSH and LH for 20 h; EGF(-): COCs were cultured without EGF for 20 h; EGF(+): COCs were cultured with EGF for 20 h; +H89: COCs were cultured with H89 for 20 h; +SB: COCs were cultured with SB203580 for 20 h; +U0126: COCs were cultured with U0126 for 20 h

When COCs were cultured without FSH and LH for 20 h, the proportion of oocytes exhibiting GVBD was less than 25%. FSH and LH significantly increased the proportion of oocytes exhibiting GVBD. This higher rate was significantly decreased by H89 or SB20580, and the suppression was counteracted by the addition of EGF (Figure 4). Treatment with U0126 also significantly suppressed the GVBD rate, whereas the addition of EGF did not overcome the negative effects.

**Figure 5:**
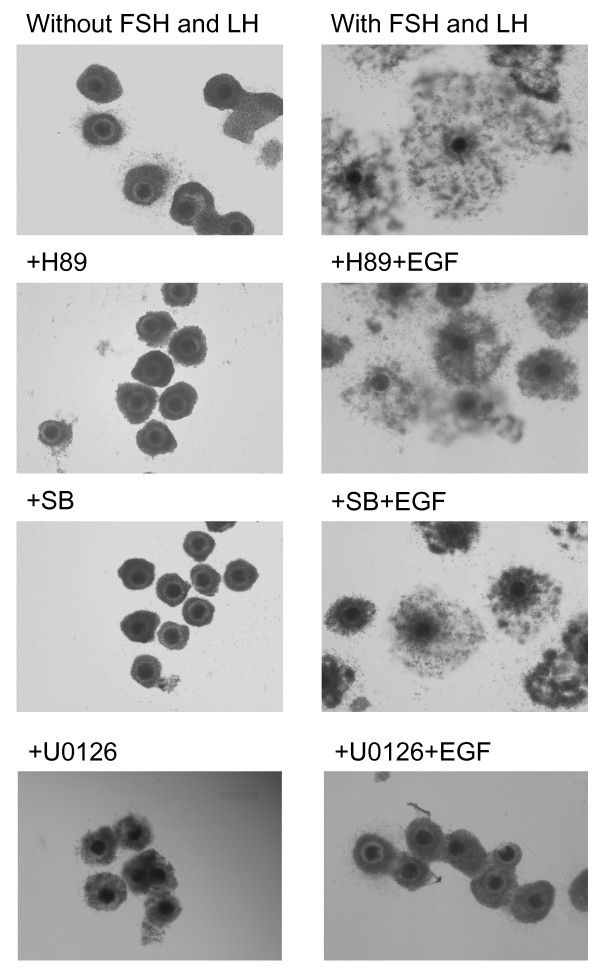
**Effect H89, SB203580 or U0126 on cumulus expansion of cultured COCs**. Without FSH and LH: COCs were cultured without FSH and LH for 40 h; With FSH and LH: COCs were cultured with FSH and LH for 40 h; +H89: COCs were cultured with FSH, LH and H89 for 40 h; +H89+EGF: COCs were cultured with FSH, LH, H89 and EGF for 40 h; +SB: COCs were cultured with FSH, LH and SB203580 for 40 h; +SB+EGF: COCs were cultured with FSH, LH, SB and EGF for 40 h; +U0126: COCs were cultured with FSH, LH and U0126 for 40 h; +U0126+EGF: COCs were cultured with FSH, LH, U0126 and EGF for 40 h.

The culture of COCs with FSH and LH for 40 h induced the full expansion of COCs (Figure. 5). The expansion was completely suppressed by treatment with H89, SB20580 or U0126 for 40 h. Although the negative effects of H89 and SB20580 were overcome by the addition of EGF, the treatment with EGF did not overcome the negative effects of U0126.

## Discussion

Recently, it has been showed that the novel paracrine/autocrine factors expressed in granulosa cells and cumulus cells by LH stimuli acted on cumulus cells to induce cumulus expansion, meiotic maturation of oocytes and ovulation in mouse [9-11]. Since the suppression of the EGF receptor tyrosine kinase activity by specific inhibitor completely suppressed cumulus expansion and meiotic maturation of oocytes during in vitro maturation of mouse COCs [9], the EGF-like factor was quite important for ovulatory process. During *in vitro *maturation of porcine COCs, the addition of EGF to maturation medium significantly elevated developmental competence of oocytes to blastocyst stage [12], indicating that investigation of the transcriptional mechanism of the EGF-like factor and TACE/ADAM17 was essential for not only ovulatory process, but also cytoplasamic maturation of oocyte.

It has been reported that during the ovulation process the PKA-dependent pathway involving the ligand activation of G protein-coupled receptors by their ligands in cumulus and/or granulosa cells induced the expression of ovulation-related genes, including *Cyp11a1 *[24], *Star *[25] and *Ptgs2 *[26]. Other reports documented that, in rat granulosa cells, FSH and LH up-regulated the phosphorylation of p38 MAPK by a PKA-independent mechanism that might be involved in Epac [27]. Furthermore, a recent report showed that FSH nongenomically activates ERK1/2 via SRC/RAS dependent pathway in rat granulosa cells [17], indicating that the activation of ERK1/2 was not directly activated by cAMP-activated PKA or p38 MAPK pathway. In this study, when porcine COCs were cultured for 2.5 h with PKA inhibitor, p38 MAPK inhibitor or MEK inhibitor, FSH- and LH-induced *Areg*, *Ereg*, and *Tace/Adam17 *mRNA expressions were significantly suppressed. In osteoblastic cells, the promoter region of the *Areg *gene had a putative CRE site, and the region was quite important for parathyroid hormone-induced *Areg *gene induction via CREB phosphorylation [28]. In rat granulosa cells, it has been reported that the phosphorylation of CREB was induced by FSH within 1.5 h [29]. Our recent study showed in rat granulosa cells that phosphorylation of CREB was induced by FSH dependent manner and the phosphorylation of CREB was essential for transcription of *Areg *mRNA via its promoter region of CRE site (Shitanaka et al., unpublished data). We also showed in granulose-specific *Erk1/2 *knockout mice that *Areg *expression level was significantly lower than that in wild-type mice [30]. It is known that CREB has the sites phosphorylated by ERK1/2 and p38 MAPK [31,32]. Thus, at early in the process, p38 MAPK and ERK1/2 might be involved in the phosphorylation of CREB, which would induce *Areg *gene expression in cumulus cells of porcine COCs.

Our previous study showed that EGFR tyrosine kinase inhibitor or TACE/ADAM17 inhibitor suppressed the phosphorylation of ERK1/2 and the meiotic resumption of oocytes [13]. In this study, the addition of EGF to PKA or p38 MAPK inhibitor-containing medium overcame the negative effects or cumulus expansion and oocyte meiotic resumption. However, when COCs were cultured with U0126, the treatment with EGF did not overcome the U0126 effects. Thus, the MEK-ERK1/2 pathway played dual roles in cumulus cells. One role is the induction of EGF-like factor and TACE/ADAM17 expression. The other is direct induction of cumulus expansion of porcine COCs. In granulosa cell specific *Erk1/2 *knockout mice, the cumulus expansion was completely suppressed via the low induction of *Ptgs2 *expression. In this study, the *Ptgs2 *expression level was suppressed by U0126, suggesting that EGF-like factor was required for the induction of cumulus expansion and oocyte meiotic resumption in porcine COCs as well as in mice.

## Conclusion

Herein, we showed that the expression of EGF-like factor and TACE/ADAM17 in cumulus cells was induced by a PKA-, p38 MAPK- and ERK1/2-dependent mechanism during *in vitro *maturation of porcine COCs. The intracellular mechanism of induction of the expression of EGF-like factor and TACE/ADAM17 further induce EGF-like factor and *Tace/Adam17 *mRNA expressions in cumulus cells via ERK1/2 activation. Thereby, the ERK1/2 maintained its activity via the EGF domain-EGFR-ERK1/2 pathway, which resulted in full cumulus expansion, and oocyte maturation during *in vitro *maturation of porcine COCs.

## Abbreviations

FSH: follicle stimulating hormone; LH: luteinizing hormone; EGF-like factor: epidermal growth factor-like factor; TACE/ADAM17: tumor necrosis factor α converting enzyme/a disinteglin and metalloptotease 17; Has2: hyaluronan synhase 2; Tnfaip6; tumor necrosis factor α-induced protein 6; Ptx3: pentraxin 3; PGE2: prostagrandin E2; PTGS2; prostagrandin synthase 2; AREG; amphiregulin; EREG: epiregulin; BTC: β-cellulin; EGFR: EGF receptor; hCG: human chorionic gonadotropin; COC: cumulus-oocyte complex; PKA: protein kinase A; p38 MAPK: p38 mitogen-activated protein kinase; PI3K: phosphatidylinositol 3-kinase; ERK: extracellular-signal regulated protein; SRC: rous sarcoma oncogene; RAS: rat sarcoma viral oncogene.

## Competing interests

The authors declare that they have no competing interests.

## Authors' contributions

YY and MS conceived of the study, participated in its design and coordination and drafted the manuscript. All authers read and approved the final manuscript.
